# CGRP receptors and TRP channels in migraine

**DOI:** 10.1186/1129-2377-16-S1-A21

**Published:** 2015-09-28

**Authors:** Pierangelo Geppetti, Silvia Benemei, Francesco De Cesaris

**Affiliations:** Dipartimento di Scienze della Salute, Università di Firenze, Florence, Italy; Centro Cefalee, Azienda Ospedaliero-Universitaria Careggi, Florence, Italy

Calcitonin gene-related peptide (CGRP) seems to play a major role in migraine mechanism. Overwhelming data report the efficacy of small molecule CGRP receptor antagonists in the treatment of migraine attacks. If some CGRP receptor (CGRP-R) antagonists could theoretically cross the blood brain barrier (BBB) one of them, olcagepant, due to its peptoid nature could not. However, the clinical development of such otherwise well tolerated compounds has been spoiled by their hepatic liability that has been considered an off-target effect. In the last few years the identification of monoclonal antibodies (Ab) for CGRP or the CGRP-R has provided an alternative strategy to maintain a good efficacy profile and circumvent the severe liver toxicity of classical small molecules CGRP-R antagonists. Indeed, data from phase-II trials are showing that the therapeutic gain between active treatment with the various anti-CGRP Abs and placebo varies from about 20% to 40% and adverse reactions are limited to irritation at the site of injection and few other minor effects. Anti-CGRP mAbs predictably may cross the BBB at a minimum extent, thus making unlikely the hypothesis that they act at sites of action within the central nervous system. This observation is associated with the prevalent localization of the complex multimeric assembly of CGRP-R in the vascular smooth muscle where they mediate the inflammatory neurogenic vasodilatation. Thus, the most parsimonious hypothesis proposes that blockade of the CGRP/CGRP-R receptor system within the cranial neurovascular system produces the desirable analgesic effect in migraine pain.

If the mechanism and the genetic background that by promoting cranial neurogenic vasodilatation generate migraine pain remain a mystery, some insights on the triggers that may activate this pathogenic pathway are now better understood. A series of agents known to provoke migraine attacks have been identified as activators of certain transient receptor potential (TRP) channels expressed by a subpopulation of peptidergic nociceptors. In particular, the subtypes ankyrin 1 (TRPA1) and vanilloid 1 (TRPV1) are activated by migraine provoking agents. Nitric oxide, umbellulone and acrolein gate TRPA1 and alcohol TRPV1. All stimuli by channel targeting generate CGRP release from perivascular terminals of cranial sensory neurons, thus producing the neurogenic effect blunted by CGRP-R antagonists and by anti CGRP mAbs. More importantly, antimigraine medicines, such as metamizole (dipyrone), propyphenazone and parthenolide exert their analgesic effect by antagonizing TRPA1.
